# δ-Tocotrienol Induces Human Bladder Cancer Cell Growth Arrest, Apoptosis and Chemosensitization through Inhibition of STAT3 Pathway

**DOI:** 10.1371/journal.pone.0122712

**Published:** 2015-04-07

**Authors:** Changxiao Ye, Wei Zhao, Minghui Li, Junlong Zhuang, Xiang Yan, Qun Lu, Cunjie Chang, Xiaojing Huang, Ji Zhou, Bingxian Xie, Zhen Zhang, Xin Yao, Jun Yan, Hongqian Guo

**Affiliations:** 1 Model Animal Research Center, MOE Key Laboratory Model Animal for Disease Study, Nanjing University, Nanjing, Jiangsu 210061, China; 2 Nanjing Drum Tower Hospital, Nanjing University Medical School, Nanjing, Jiangsu 210008, China; 3 Nanjing Urology Research Center, Nanjing, Jiangsu 210008, China; 4 Department of Respiratory Medicine, The first affiliated hospital of Nanjing Medical University, Nanjing, Jiangsu 210029, China; 5 Zhejiang Provincial Key Lab for Technology and Application of Model Organisms, School of Life Sciences, Wenzhou Medical College, Wenzhou, Zhejiang 325035, China; Thomas Jefferson University, UNITED STATES

## Abstract

Vitamin E intake has been implicated in reduction of bladder cancer risk. However, the mechanisms remain elusive. Here we reported that δ-tocotrienol (δ-T3), one of vitamin E isomers, possessed the most potent cytotoxic capacity against human bladder cancer cells, compared with other Vitamin E isomers. δ-T3 inhibited cancer cell proliferation and colonogenicity through induction of G1 phase arrest and apoptosis. Western blotting assay revealed that δ-T3 increased the expression levels of cell cycle inhibitors (p21, p27), pro-apoptotic protein (Bax) and suppressed expression levels of cell cycle protein (Cyclin D1), anti-apoptotic proteins (Bcl-2, Bcl-x_L_ and Mcl-1), resulting in the Caspase-3 activation and cleavage of PARP. Moreover, the δ-T3 treatment inhibited ETK phosphorylation level and induced SHP-1 expression, which was correlated with downregulation of STAT3 activation. In line with this, δ-T3 reduced the STAT3 protein level in nuclear fraction, as well as its transcription activity. Knockdown of SHP-1 partially reversed δ-T3-induced cell growth arrest. Importantly, low dose of δ-T3 sensitized Gemcitabine-induced cytotoxic effects on human bladder cancer cells. Overall, our findings demonstrated, for the first time, the cytotoxic effects of δ-T3 on bladder cancer cells and suggest that δ-T3 might be a promising chemosensitization reagent for Gemcitabine in bladder cancer treatment.

## Introduction

Bladder cancer is a major clinical problem worldwide. It is the second most common type of urinary tract cancer in the developed countries, with the estimation of 74,690 new cases and 15,580 deaths in USA in 2014 [1]. Unfortunately, bladder cancer is also one of the most recurrent and expensive malignancies, with four billion US dollar annual cost on bladder cancer patients in USA during 2010 [2–4]. Surgical resection, radiation and chemotherapy are common therapeutic approaches for bladder cancer. However, different side effects are associated with each treatment and some cancer cells eventually become drug resistant. Therefore, it is imperative to develop novel strategies to combat bladder cancer, including complementary therapies that can be used in combination with current treatments.

Vitamin E intake has been inversely related to bladder cancer risk among older individuals or heavy smokers from multiple epidemiologic studies [5,6]. Both tocopherols (TP) and tocotrienols (T3) belong to the vitamin E family, and each subfamily is composed of four isomers: α-, β-, δ- and γ. The main difference between TP and T3 is the structure of their side chains, with farnesyl for T3 and saturated phytyl for TP [7–9]. Compared to TPs, which are commonly found in the leaves and seeds of most plants, T3s are less abundant and mainly found in palm oil and rice bran. Two clinical trials, the Women Health Study (WHS) trial and the Selenium Vitamin E and Prostate Cancer Chemoprevention Trial (SELECT), were carried out to investigate the cancer prevention property of α-TP [10,11]. Neither trial showed significant effect of α-TP against lung, breast and colon cancer in women and prostate cancer in men. Therefore, different T3 isomers have evoked more research attention recently, due to their potential application as non-toxic dietary anti-cancer agent [12–14]. Among them, δ-T3 showed strong potency against various types of cancers, including pancreatic, colorectal and breast cancer [15–17]. However, whether δ-T3 possesses anticancer activity against bladder cancer has not yet been explored.

The activation of Signal Transducer and Activator of Transcription 3 (STAT3) is frequently detected in various cancer types, including bladder cancer [18]. The phosphorylation of 705 tyrosine residue in STAT3 protein, which is a crucial event for its activation, leads to form STAT3 homodimers and translocation into the nuclei. Nuclear localized STAT3 dimer binds to the promoters of various target genes and regulates their transcriptions, which are involved in cancer cell proliferation, survival and invasion [19]. Moreover, it is reported that ultraviolet induced cell apoptosis can be repressed by STAT3 activation; whereas STAT3 inhibition induces Caspase dependent apoptosis and inhibits cell migration and angiogenesis in cancer cells [20,21]. Recent study further revealed that constitutively activated STAT3 in urothelial cells accelerates the progression into muscle-invasive bladder cancer, indicating that STAT3 plays a critical role in bladder cancer development [22].

In this study, we observed the stronger cytotoxicity of δ-T3 on human bladder cancer cell lines than non-malignant immortalized urothelial cells. Mechanistically, we showed that δ-T3 inhibited ETK activation and up-regulated SHP-1 expression, which is correlated with the suppression of STAT3 signaling pathway. We also demonstrated low dose of δ-T3 enhanced the sensitivity of bladder cancer cells to chemotherapeutic agent--Gemcitabine.

## Materials and Methods

### Reagents and cell lines

All chemicals and reagents were purchased from Sigma-Aldrich (St. Louis, MO) unless otherwise specified. α-, γ-, δ-T3 and α-tocopherol (α-TP) were kindly supplied by Davos Life Science Ltd (Synapse, Singapore). Gemcitabine was from Eli Lilly Company (Indianapolis, IN). Bcl-2, Bcl-x_L_, Mcl1, PARP, pro-Caspase-3, pSTAT3(Y705), pETK(Y40), STAT3, and SHP-1 antibodies were purchased from Cell Signaling Technology, Inc. (Danvers, MA). ETK, p21 and p27 antibodies were from BD Biosciences (San Jose, CA). β-Actin antibody was purchased from AbMax Biotechnology Company (Beijing, China). Bax antibody was purchased from Santa Cruz Biotechnology (Santa Cruz, CA). The non-malignant immortalized urothelial cell line SV-HUC-1, bladder cancer cell lines T24, 5637, J82 and UMUC-3 were obtained from Cell Bank, Type Culture Collection, Chinese Academy of Sciences (Shanghai, China). All cell lines were cultured in RPMI 1640 supplemented with 10% FBS, 100 units/ml penicillin, and 100 μg/ml streptomycin. Cells are cultured in at 37°C in a humidified atmosphere of 95% air and 5% CO_2_. As for siRNA interfering assay, SHP-1 targeting siRNA: 5’-GAGAACGCUAAGACCUACAtt-3’ and control siRNA: 5’-UUCUCCGAACGUGUCACGUtt-3’ were transfected into cancer cells using Lipofectamine 2000 (Invitrogen), respectively.

### Cell viability assay

For cell viability study, 1.5×10^3^ bladder cancer cells were plated into each well of a 96-well plate. The cells were then treated with different concentration (50, 100, 150, 200 μM) of the vitamin-E isomers for 24, 48 and 72h. After treatment, 10 μl of 5 mg/ml MTT solution was added into each well and the cells were incubated at 37°C for 3 h. The formazan crystals were then re-suspended in 100 μl DMSO and the absorbance at 490 nm was measured. Each experiment was repeated three times and the growth curves showed the means and standard deviation.

### Colony formation assay

After 14-day incubation of 100 μM α-TP, α-T3, γ-T3 and δ-T3, colonies were fixed with 100% methanol, and stained with 0.5% crystal violet. Only colonies with >50 cells were counted. Each treatment was repeated in triplicate.

### Flow cytometry analysis

Cells were incubated with indicated concentrations of δ-T3 for 48 hr, before samples were fixed by 70% ethanol. Cells were incubated in 10 mg/ml RNase A containing PBS for 30 min at 37°C, followed by addition of 1 mg/ml propidium iodine (Sigma). Afterwards, cells were analyzed using a fluorescence-activated cell sorting (FACS) Calibur flow cytometer (BD FACS Calibur, BD Biosciences, San Jose, CA).

### Apoptosis analysis

Annexin V/propidium iodide (PI) staining was performed using Alexa Fluor 488 Annexin V/Dead Cell Apoptosis Kit (Invitrogen, Cat# V13245, Inc., Carlsbad, CA, USA) according to the manufacturer's guidelines. Briefly, 1x10^6^ cells were trypsinized, followed by the resuspension in 100 μl of binding buffer and incubated with 1.0 μl of PI and 5.0 μl of Annexin V-fluorescein isothiocynate for 15 min in the dark at room temperature. Afterwards, cells were detected using FACS Calibur Instrument (Becton Dickinson) and analyzed using Flowjo 7.6 software.

### Real-time reverse-transcription polymerase chain reaction analysis

Total RNA was extracted with Trizol (Invitrogen). The expression levels of *bcl2*, *bclxl and mcl1* genes were detected by quantitative reverse-transcription polymerase chain reaction (qRT-PCR). The primers are listed as follows: *bcl2*: forward, 5’-CGTACAGTTCCACAAAGGCA-3’ and reverse, 5’-ATGTGTGTGGAGAGCGTCAA-3’; *bclxl*: forward, 5’-TTCAGTGACCTGACATCCCA-3’ and reverse, 5’-TTCAGTGACCTGACATCCCA-3’; *mcl1*: forward, 5’-TCCCTGGAGAAGAGCTACG-3’ and reverse, 5’-GTAGTTTCGTGGATGCCACA-3’; *β-actin* was used as an internal control. The primers for *β-actin* were 5’-AGCGAGCATCCCCCAAAGTT-3’ and 5’-GGGCACGAAGGCTCATCATT-3’. qPCR was performed using the SYBR Green (TaKaRa Biotechnology Co. Ltd, Dalian, China) dye detection method on ABI StepOne Sequence Detection System under default conditions: 95°C for 10 min, and 40 cycles of 95°C for 5 s and 55°C for 31 S. Comparative Ct method was used for quantification of the transcripts.

### Western blotting assay

Cells were lysed in a RIPA buffer containing protease inhibitor cocktail tablet (Roche Diagnostics, Indianapolis, IN) and phosphatase inhibitor cocktail I (Sigma). Cell lysates (20 μg) was resolved on SDS-PAGE, and transferred onto PVDF membrane. After blotting in 5% non-fat dry milk in phosphate-buffered saline containing 0.1% Tween-20 (PBST), the membranes were incubated with primary antibodies at 1:500 to 1,000 dilutions in PBST or 5% BSA overnight at 4°C, according to manufacturers’ instructions. The blots were washed and incubated with horseradish peroxidase-conjugated secondary antibodies for 1 hr, and finally detected by ECL reagent (Thermo Scientific).

### Luciferase activity assay

The effect of δT3 on STAT3 responsive element containing promoter (STAT3-Luc) activity was analyzed using a luciferase assay. T24 cells (2.5 × 10^5^ per well) were seeded in 24-well plates. After overnight culture, the cells were transfected by lipofectamine 2000 (Invitrogen) with 0.5 μg of STAT3-Luc and 1 ng of TK-Renilla luciferase plasmid. After 24 h of transfection, the cells were incubated with δT3 for 24 h and harvested. Luciferase activity was measured using the dual luciferase assay system (Promega) and detected using the Victor microplate reader (Perkin-Elmer).

### Nuclear extracts preparation

T24 cells were plated in 10 cm dish, followed by the treatment with 150 μM δ-T3 for 24 h. Cells were harvested by trypsinization and washed twice with ice-cold PBS. The cell pellet was gently resuspended in 4X volumes of ice-cold hypotonic lysis buffer (10 mM HEPES, pH 7.9, 1.5 mM MgCl_2_, 10 mM KCl, 0.3% NP-40, 0.1 mM EDTA, 0.1 mM EGTA, 0.5 mM DTT) with protease inhibitors (Roche), and kept on ice for 30 min. Cells were kept in the buffer till they have swollen. The cell lysates were centrifuged for 10 min at 10,000×g. The supernatants were cytosolic extracts. Resuspend the precipitate (nuclear fraction) in 4X volumes of strong protein lysis buffer (10 mM HEPES, 1.5 mM MgCl_2_, 420 mM NaCl, 0.2 mM EDTA, 0.1 mM EGTA, 25% glycerol, 0.5 mM DTT) with protease inhibitor for 15 min. Supernatant was collected as nuclear protein by centrifugation at 12,000 rpm for 30 min at 4°C.

### Chromatin immunoprecipitation (ChIP)

ChIP assay was performed by using ChIP assay kit (Cat. 17–295, Millipore), according to the manufacturer’s instructions. After nuclear extracts were isolated as shown above, antibody against STAT3 (CAT# 9139, Cell Signaling Technology) was used to immunoprecipitate chromatin in nuclear fractions, whereas a nonspecific IgG antibody was used as a technical negative control. Measurements were made in triplicate and done by Applied Biosystems StepOne Real-Time PCR System (AppliedBiosystems, Carlsbad, CA, USA). The sequences of primers used for the ChIP–qPCR analysis of the *bclxl gene* promoter are as follows: Bcl-xL-primer 2F 5’-CTGGGTTCCCTTTCCTTCCA-3’, Bcl-xL-primer 2R 5’-TCCCAAGCAGCCTGAATCC-3’ [23]; Bcl-xL-primer 1F 5’-CCCTTGCAGCTAGTTTTCTA-3’, Bcl-xL-primer 1R 5’-TGAATTCTGAGGCCAAGGGAA-3’; Bcl-xL-primer NCF 5’- TGCCAGGCCTTCGCTCAA-3’, Bcl-xL-primer NCR 5-CAGATAAACTCTGTCCACAGA-3’. Primer set 1 is 954 bp downstream from transcription start site, whereas primer set 2 is 1,277 bp downstream from TSS, covering a conserved STAT3 binding motif predicted by website (www.dcode.org). Primer NC is localized at 1,880 bp downstream from the last exon, serving as a negative control.

### Statistical analysis

All numeric values are represented as the mean ± SD. The Student unpaired t-test was used for statistical analysis. Significance was set as *P* < 0.05.

## Results

### Cytotoxic effect of vitamin E isomers on bladder cancer cells

**Fig 1:**
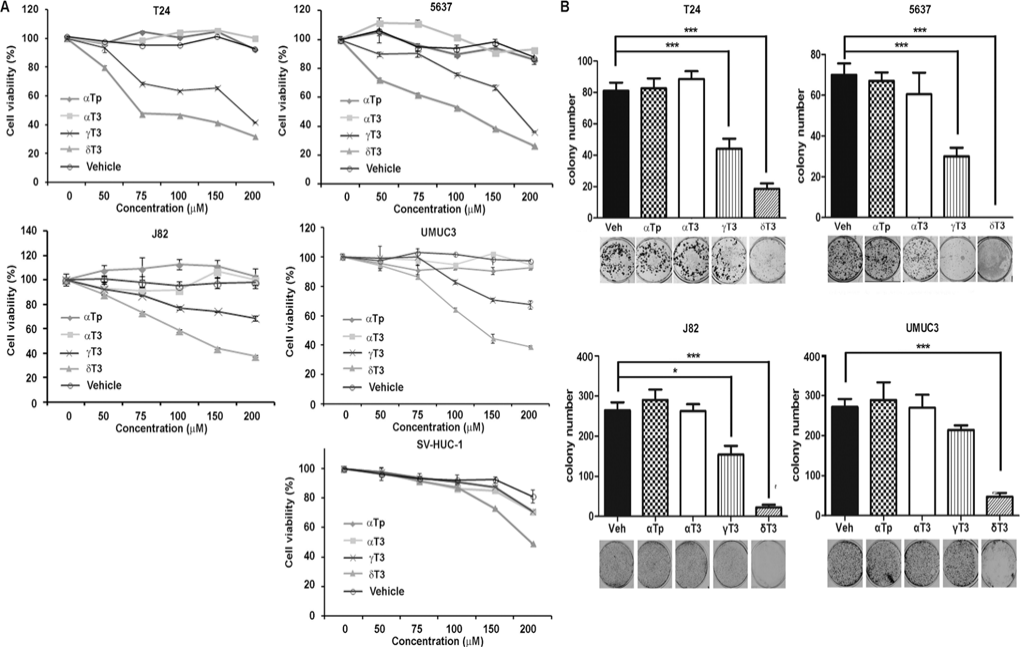
Effects of vitamin E isomers on bladder cancer cells. (A) Human bladder cancer cells T24, 5637, J82 and UMUC-3, as well as non-malignant human urothelial SV-HUC-1 cells were treated with each vitamin E isomers (α-TP, α-T3, γ-T3 and δ-T3) ranging from 0 to 200 μM for 72 h, and cell viability were detected by MTT assay. (B) Colony formation assay of T24 5637, J82 and UMUC-3 cells with 100 μM α-TP, α-T3, γ-T3 and δ-T3 for 14 days. Vertical bars indicate the mean cell count ±SD in each treatment group. *, *P* < 0.05; ***, *P* < 0.001, compared to vehicle treatment group.

In order to examine which vitamin E isomers have cytotoxic effects on human bladder cancer cells *in vitro*, we treated four commonly used human bladder cancer cell lines, T24, 5637, J82 and UMUC-3. These four cell lines contain various degrees of genetic complexity, covering the common genetic mutations found in human bladder cancer samples. These include the inactivation of TP53 and Rb1, activation of K-Ras and H-Ras or loss of Pten (www.atcc.org). The results showed that both γ- and δ-T3 displayed anti-cancer effects in a concentration-dependent manner, whereas α-T3 as well as α-TP did not show any cytotoxic effects within the range of dosages that we tested (Fig 1A). To test cytotoxic effects of vitamin E isomer on normal bladder cells, we also treated SV-HUC-1, an immortalized non-malignant urothelial cell with the vitamin E isomers. Of note, γ- and δ-T3 exerted less toxicity on non-malignant bladder epithelial cell (Fig 1A). In addition, treatment of δ-T3 and γ-T3 also significantly decreased colony numbers, compared to α-T3 and α-TP (Fig 1B). Using the MTT assay and colony formation assay, we demonstrated that the order of inhibitory effect by vitamin E isomers is δ-T3 > γ-T3>> α-T3 and α-TP in all of four human bladder cancer cell lines; therefore, δ-T3 was chosen for further study.

### δ-T3 causes G1 arrest and apoptosis

**Fig 2:**
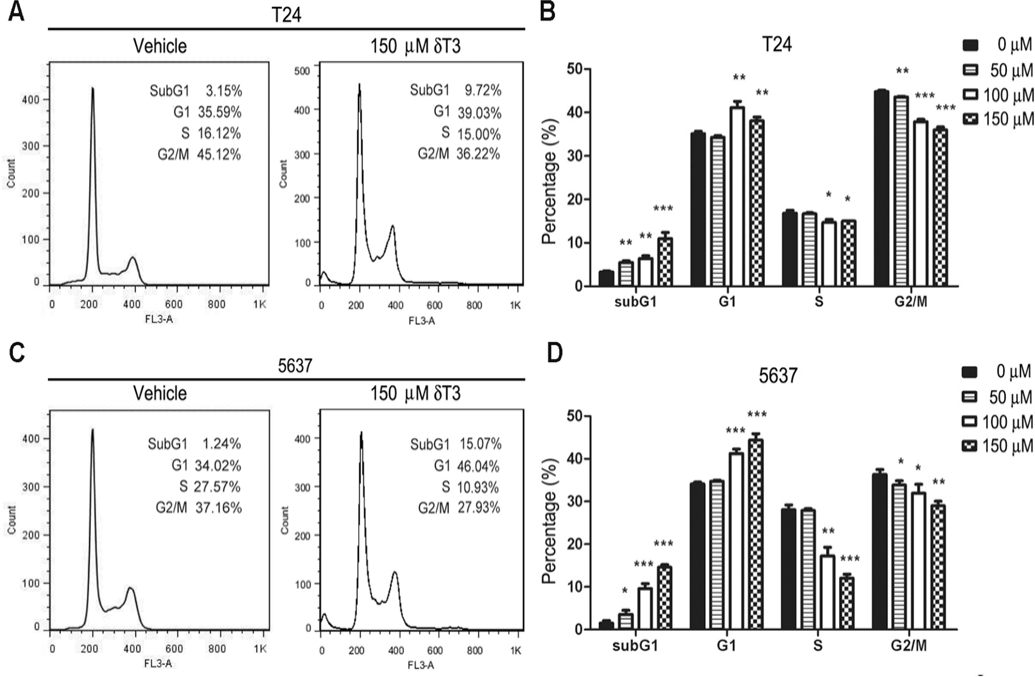
δ-T3 induced cell cycle arrest in T24 (A, B) and 5637 cells (C, D) bladder cancer cells. Cells were treated by δ-T3 ranging from 0–150 μM for 48 h. Cell cycle distribution and Sub-G1 ratios were assessed by flow cytometry. *, *P* < 0.05; **, *P* < 0.01; ***, *P* < 0.001.

**Fig 3:**
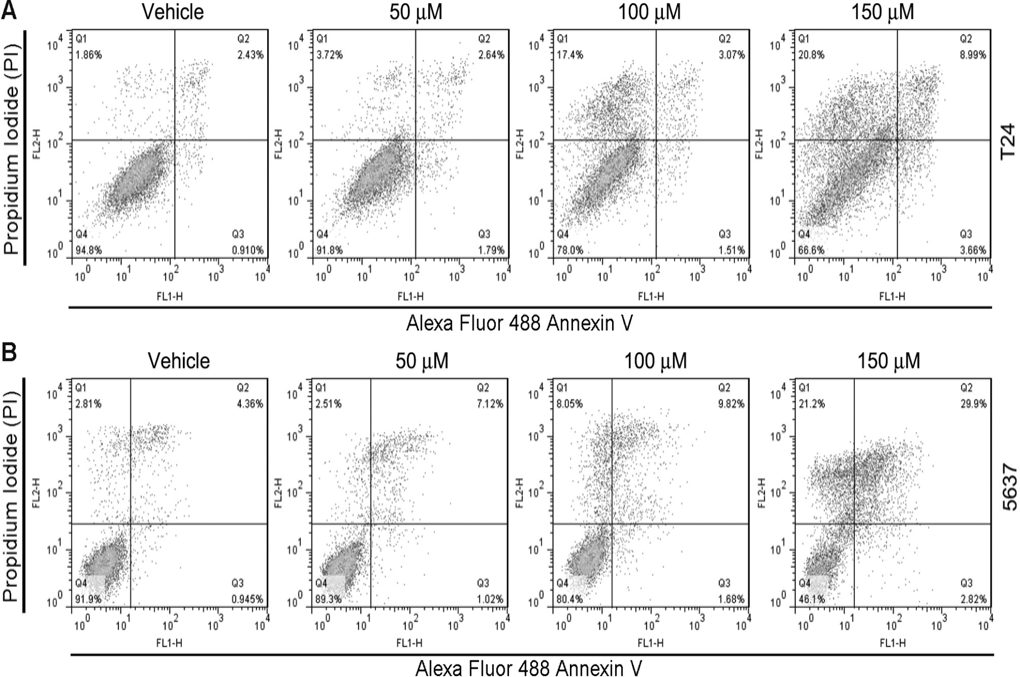
δ-T3 induces apoptosis in bladder cancer cells. Annexin V/PI analysis showed that δT3 induced apoptosis in T24 (A) and 5637 (B) bladder cancer cells, as compared to vehicle treated control cells.

To further demonstrate the anti-cancer effects of δ-T3, we analyzed cell cycle distribution by flow cytometry (Fig 2A–2D). T24 and 5637 cells were treated with different concentrations of δ-T3 (0, 50, 100 and 150 μM) for 48 h. Flow cytometry analysis showed that δ-T3 treatment (≥ 100 μM) induced G1 phase arrest and reduction of cell population in S phase. Furthermore, apoptotic cell population (sub-G1) increased in a concentration-dependent manner (Fig 2A–2D). To further characterize the induction of apoptosis, we performed Annexin V/PI staining assay. As shown in Fig 3, δ-T3 treatment induced apoptotic index in both of T24 (Fig 3A) and 5637 (Fig 3B) cells in a concentration-dependent manner.

### δ-T3 induces cell cycle inhibitors and down-regulates pro-survival pathway

**Fig 4:**
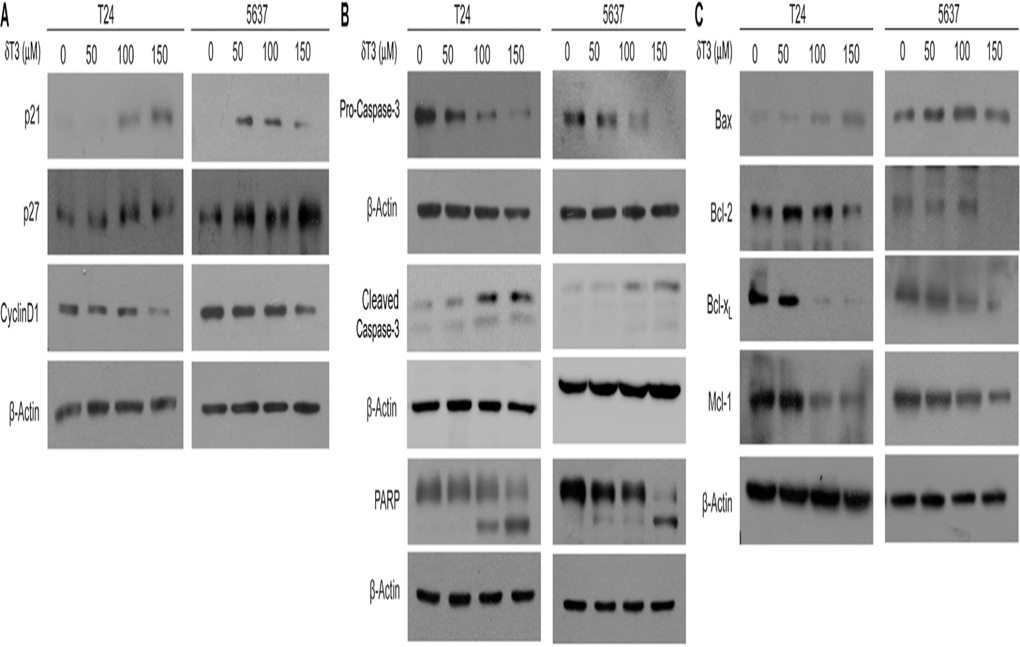
δ-T3 treatment changes protein expression levels involved in cell cycle arrest and apoptosis. Western blotting analysis of the cell cycle (A), apoptosis (B) and other apoptosis-related (C) protein levels in T24 and 5637 cells, upon the δ-T3 treatment for 24 h. β-Actin was used as the loading control.

Western blotting analysis was carried out to further examine the expression levels of proteins involved in cell cycle and apoptosis. After 24 h treatment with δ-T3 at different concentrations in T24 and 5637 cells, we observed the increased expression levels of cell cycle inhibitors, p21^Waf1/Cip1^ and p27^Kip1^, and decreased expression levels of Cyclin D1 (Fig 4A). Moreover, δ-T3 treatment also activated pro-Caspase-3, and resulted in the PARP cleavage (Fig 4B). Since the activation of Caspase-3 may be via mitochondrial pathway, we tested apoptosis-related proteins on the mitochondria. As shown in Fig 4C, the expression level of the pro-apoptotic protein, Bax was enhanced; on the other hand, anti-apoptotic proteins, Bcl-2, Bcl-x_L_ and Mcl-1 were repressed upon the δ-T3 treatment. As shown in S1 Fig, the cleavage of apoptotic markers (Caspase-3 and PARP), the induction of pro-apoptotic Bax protein, as well as reduction of anti-apoptotic proteins were also confirmed in another two bladder cancer cell lines (J82 and UMUC-3).

### δ-T3 dephosphorylated ETK and upregulated SHP-1 to suppress STAT3 signaling

**Fig 5:**
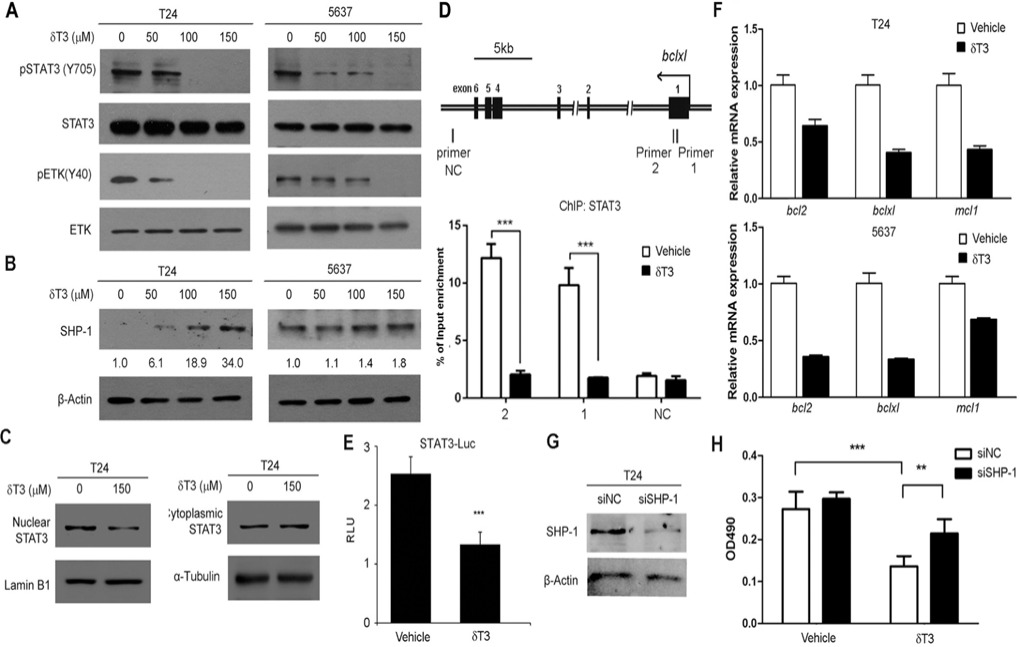
δ-T3 suppresses STAT3 signaling pathways in human bladder cancer cells. Western blotting analysis of the STAT3/ETK signaling pathway-related (A) and SHP-1 (B) protein levels in T24 and 5637 cells under the δ-T3 treatment for 24 h. Western bands were quantified by Image J software and the digits shown below the upper panel were the relative STAT3 expression levels normalized by loading controls. (C) Reduction of nuclear STAT3 protein level upon the treatment of 150 μM δ-T3 in T24 cells. LaminB1 was used as a nuclear loading control. Tubulin was used as a cytoplasmic loading control. (D) Genomic structure of *bclxl* gene was shown, with the labels of three primer sets for ChIP assay. Primer set 1 and 2 contain STAT3 binding element; whereas primer set 3 (NC) serves as negative control. STAT3 occupancy in the *bclxl* promoter in bladder cancer cell line T24 treated with 150 μM δ-T3 or vehicle for 18 h were assayed by ChIP assay. Input DNA and immunoprecipitated DNA were analyzed by qPCR analyses using primer sets depicted above and normalized by IgG control. The error bar indicates the means ± SD. ***, *P <* 0.001. (E) δ-T3 treatment reduced the STAT3 downstream target genes (*bcl2*, *bclxl* and *mcl-1*) expression at mRNA level. (F) Luciferase activity analysis of STAT3-Luc upon the treatment of 150 μM δ-T3 in T24 cells for 24 h. TK-Renilla luciferase plasmid was used as internal control. (G) Knockdown efficiency of SHP-1 in T24 cells by Western blotting assay. β–Actin was used as internal control. siNC, negative control siRNA. siSHP-1, siRNA targeting to SHP-1. (H) T24 cells were treated with siRNA to SHP-1 or siNC, followed by treatment with δ-T3 or vehicle. Cell viability was tested by MTT assay. **, *P* < 0.01; ***, *P* < 0.001.

To further dissect the effects of δ-T3 on downstream signaling, we analyzed STAT3 signaling pathway, which is one of the major anti-apoptotic pathways, conferring the survival advantage and chemo-resistance of bladder cancer cells against various chemotherapeutic agents [18–21]. We found that δ-T3 suppressed the phosphorylation level of STAT3(Y705) in a concentration-dependent manner in both of T24 and 5637 bladder cancer cell lines (Fig 5A). Activation of STAT3 is regulated by upstream kinases and phosphatases. In bladder cancer, epithelial and endothelial tyrosine kinase (ETK) is frequently overexpressed [24]. As a non-receptor tyrosine kinase, ETK activation/expression is required for STAT3 activation in cancer cells. In line with this, we observed that the active form of ETK (phosphorylation at Y40) was reduced with δ-T3 treatment, starting from the concentration of 50 μM in both T24 and 5637 cells. Furthermore, δ-T3 strikingly induced the expression level of protein tyrosine phosphatase SHP-1, which functions as a negative regulator for STAT3 activation (Fig 5B). Nuclear translocation of STAT3 is essential for its function as a transcription factor. The nuclear and cytosol fractions of protein lysate from the δ-T3 treated cells were separated. Upon the δ-T3 treatment, STAT3 protein level in nuclei was decreased in T24 cells (Fig 5C). To further confirm the reduction of nuclear STAT3 occupancy on its target genes, we performed ChIP assay using antibody against STAT3. As shown in Fig 5D, δ-T3 significantly reduced the recruitment of STAT3 onto its binding sites in *bclxl* locus by 5.55 folds (primer set 1) and 5.93 folds (primer set 2), whereas negative control primer set (NC) did not shown any difference. In concert with this result, the transcriptional activity of STAT3-responsive promoter was significantly repressed by δ-T3 treatment in a luciferase reporter assay (Fig 5E). Moreover, qRT-PCR results revealed that three STAT3 direct target genes (*bcl2*, *bclxl* and *mcl1*) were downregulated at mRNA levels in both two bladder cancer cell lines (Fig 5F), indicating that the reduction is mainly due to the transcriptional regulation. To examine whether SHP-1 is essential for STAT3 activation induced cell survival, we knocked down endogenous SHP-1 by RNA interference (Fig 5G) and found that its depletion partially abrogated δ-T3 induced toxicity to T24 cells (Fig 5H). This also confirmed that δ-T3 induced bladder cancer cell proliferation inhibition partial through SHP-1 induction. Taken together, our data demonstrated that δ-T3 treatment decreased the phosphorylation level along with the nuclear translocation of STAT3 protein, resulting in the transcriptional reduction of its downstream target genes in human bladder cancer cells.

### Low concentration of δ-T3 potentiated the apoptotic effect of gemcitabine on T24 bladder cancer cells

**Fig 6:**
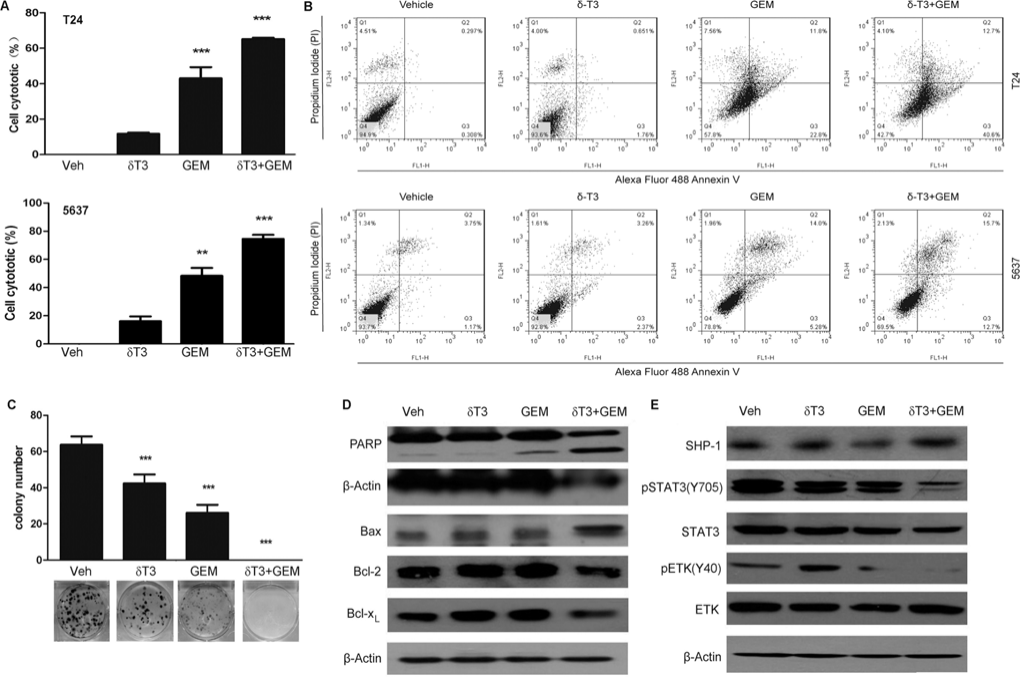
Low concentration of δ-T3 enhanced the anti-cancer effects of Gemcitabine (GEM) on bladder cancer cell growth. (A) T24 and 5637 cells were incubated for 48 h in the presence of 25 μM δ-T3 and/or 0.08 μM GEM. Then, the percentage of cell viability was determined by MTT assay. (B) T24 and 5637 cells were cultured for 48 h in the absence or presence of 25 μM δ-T3 and/or 0.08 μM GEM, respectively. Apoptotic rates were analyzed by Annexin V/PI staining assay. (C) Combined treatment with GEM (0.08 μM) and δ-T3 (25 μM) for 14 days completely eliminated the colony formation capacity of T24 cells. (D) Western blotting analysis of the apoptosis-related and STAT3 signaling-related protein levels in T24 cells, treated with δ-T3 and/or GEM for 48 h. (E) Western blotting analysis of the STAT3 signaling-related protein levels in T24 cells, treated with δ-T3 and/or GEM for 24h. **, *P* < 0.01; ***, *P* < 0.001.

Gemcitabine is the first-line chemotherapy for bladder cancers [25,26]. Therefore, we examined whether low concentration of δ-T3 can increase the sensitivity of bladder cancer cells to Gemcitabine. MTT assay showed that the treatment of 25 μM δ-T3 enhanced the cytotoxic effect of Gemcitabine on T24, 5637, J82 and UMUC-3 cell lines (Fig 6A and S2 Fig). Flow cytometry data by Annexin V/PI costaining revealed that combined treatment increased the apoptotic effect, compared to Gemcitabine treatment alone. The apoptotic rate increased from 34.6% and 19.28% in Gemcitabine-treated cells to 53.3% and 28.4% in the combined treated T24 and 5637 cells, respectively (Fig 6B). Colony formation assay also showed that treatment of Gemcitabine plus δ-T3 significantly suppressed colony formation capacity of T24 cancer cells (Fig 6C). As shown in Fig 6D, co-treatment with low concentrations of δ-T3 and Gemcitabine strikingly induced Bax and repressed Bcl-xL and Bcl-2, accompanied by the presence of PARP cleavage. Consistently, we also found that co-treatment reduced phosphorylation level of ETK, induced SHP-1 expression and inhibited their downstream STAT3 activation (Fig 6E).

## Discussion

To our knowledge, this is the first report about the cytotoxic effect of δ-T3 on human bladder cancer cells. In comparison with other vitamin E isomers, our results showed that both δ- and γ-T3 are potent inhibitors in bladder cancer cell proliferation. In contrast, α-T3 and α-TP do not have anti-cancer effects against bladder cancer cells under the same experimental settings. Although both δ- and γ-T3 have significant cytotoxicity against bladder cancer cells, δ-T3 appears to be more potent than γ-T3, suggesting that it is more bioactive. This is consistent with the observations when δ-T3 is used in the treatments of other cancers, including lung and pancreatic cancer [13,15,27]. Moreover, in comparison to γ-T3, it is less known about the anti-cancer effects of δ-T3. Therefore, it would be interesting to examine whether δ-T3 could potentially be used as one of the leading vitamins for chemoprevention and treatment of bladder cancer.

In this report, we further demonstrated that δ-T3 inhibited STAT3 signaling pathway, which plays a critical role in cancer cell proliferation and survival. It is reported that STAT3 are frequently activated in various cancer types, including bladder cancer. By using phosphorylation level of STAT3 at Y705 as a surrogate marker, Lin and colleagues found that STAT3 is overactivated in 19% human bladder cancer tissues (*n* = 100) [28]. Weissmann and colleagues found that pSTAT3 is elevated in the cancer stem cell population in UBC specimens, suggesting that activation of STAT3 plays a role in the maintenance of cancer cell stemness [29]. In line with these, transgenic expression of STAT3 in basal cells of mouse bladder epithelium accelerated the progression from carcinoma *in situ* to invasive bladder cancer under carcinogen nitrosamine treatment, compared to wild-type mice under the same treatment [22]. Of note, interruption of STAT3 signaling pathway by either knockdown STAT3 or overexpression of dominant negative form of STAT3 induces cell growth arrest and apoptosis [30], indicating that targeting STAT3 signaling is a promising therapeutic approach for bladder cancer patients.

Activation of STAT3 is regulated by upstream kinases and phosphotases. ETK, also known as BMX, is a member of the Tec family of non-receptor tyrosine kinase. It contains several critical domains including the Plekstrin homology (PH), the Tec homology (TH), the Src homology SH3, SH2, and the SH1 kinase domain. Elevated expression of ETK has been detected in skin hyperplasia and prostate cancer [31,32]. Recently, ETK was suggested to be used as a biomarker to predict the survival rate of patients with cystectomy in bladder cancer [24]. Its oncogenic function has been related with its interaction with and activation of STAT3 [24,33]. As overexpressed in glioblastoma stem cells (GSCs), ETK activates STAT3 to maintain self-renewal and tumorigenic potential of GSCs [34]. Moreover, knockdown of ETK suppresses the activation of STAT3 in two bladder cancer cell lines T24 and UM-UC-3 [24], indicating that ETK functions upstream of STAT3 in bladder cancer cells.

In addition, SHP-1 is a non-transmembrane PTP that functions as a negative regulator of the STAT3 pathway [35]. As a tumor suppressor, the promoter of SHP-1 is frequently hypermethylated in leukemia and lymphoma cells [36]. We first reported that δ-T3 induces SHP-1 in a dose-dependent manner in bladder cancer cells. Since γ-T3 also induce SHP-1 expression to inhibit STAT3 signaling in myeloma and hepatocellular carcinoma cells [36,37], both γ- and δ-T3 may function in a similar way to inhibit STAT3 signaling by inducing SHP-1 expression. Consistent with the repression of STAT3 pathway, which are highly relevant to bladder carcinogenesis [22], the suppression of STAT3 was further confirmed by the reduction of its downstream targets, such as Bcl-2, Bcl-x_L_ and Mcl-1, at both mRNA and protein levels. Cellular fractionation with Western blotting and luciferase assay using STAT3-Luc plasmid confirmed that δ-T3 affects STAT3 activation through inhibition of its nuclear translocalization to induce its target genes. These data further substantiated the inhibition of STAT3 signaling pathway by δ-T3.

Gemcitabine is commonly used chemotherapeutic drug for the treatment of bladder cancer. We found that low dose of δ-T3 enhanced Gemcitabine-induced apoptosis. When compared to Gemcitabine treatment alone, co-treatment using δ-T3 and Gemcitabine strikingly suppressed the activation of ETK, STAT3 and induction of SHP-1. This supports the notion that δ-T3 potentiates Gemcitabine mediated apoptosis through inducing the cells into SubG1 population followed by cleavage of PARP. Consistently, Kanai *et al* found that vitamin E succinate induced bladder cancer cell apoptosis and enhanced chemosenstivity to paclitaxel [38]. Considering that bladder cancer grows outward to the bladder cavity, the instillation of therapeutic agents can be easily retained in the bladder, which might increase the efficacy of drugs. Gemcitabine plus δ-T3-based regimen could be a very promising chemotherapy approach to test *in vivo*.

In conclusion, we demonstrated for the first time that δ-T3 is a potent agent against human bladder cancer cells. Mechanistically, through inhibiting ETK activation and inducing SHP-1 expression, δ-T3 suppresses STAT3 pathways. Finally, since it enhances Gemcitabine-induced cancer cell apoptosis, δ-T3 could be explored as a chemo-sensitizer in bladder cancer therapy.

## Supporting Information

S1 Fig δ-T3 treatment induced apoptotic-associated protein changes in J82 and UMUC-3 cells. The cleavage of Caspase-3 and PARP (A), as well as the induction of pro-apoptotic Bax protein level and reduction of anti-apoptotic Bcl-2, BclxL and Mcl-1 protein levels were detected in J82 and UMUC-3 cells, upon the δ-T3 treatment for 24 h. β-Actin was used as the loading control.
(TIF)

S2 Fig Low concentration of δ-T3 enhanced the anti-cancer effects of Gemcitabine (GEM) on J82 and UMUC-3 bladder cancer cells. J82 and UMUC-3 cells were incubated for 48 h in the presence of 25 μM δ-T3 and/or 0.08 μM GEM. Then, the percentage of cell viability was determined by MTT assay. **, *P* < 0.01; ***, *P* < 0.001.
(TIF)

## References

[1] Siegel R, Ma J, Zou Z, Jemal A. Cancer Statistics, 2014, CA Cancer J Clin. 2014; 64: 9–29. 10.3322/caac.21208 24399786

[2] Yeung C, Dinh T, Lee J. The health economics of bladder cancer: an updated review of the published literature. Pharmacoeconomics 2014; 32:1093–104. 10.1007/s40273-014-0194-2 25056838

[3] Svatek RS, Hollenbeck BK, Holmäng S, Lee R, Kim SP, Stenzl A, et al The Economics of Bladder Cancer: Costs and Considerations of Caring for This Disease. Eur Urol. 2014; 66: 253–262. 10.1016/j.eururo.2014.01.006 24472711

[4] Castillo-Martin M, Domingo-Domenech J, Karni-Schmidt O, Matos T, Cordon-Cardo C. Molecular pathways of urothelial development and bladder tumorigenesis. Urol Oncol. 2010; 28: 401–408. 10.1016/j.urolonc.2009.04.019 20610278

[5] Brinkman M, Karagas MR, Zens MS, Schned A, Reulen RC, Zeegers MP. Minerals and vitamins and the risk of bladder cancer: results from the New Hampshire Study. Cancer Causes Control 2010; 21: 609–619. 10.1007/s10552-009-9490-0 20043202 PMC2839516

[6] Michaud DS, Pietinen P, Taylor PR, Virtanen M, Virtamo J, Albanes D. Intakes of fruits and vegetables, carotenoids and vitamins A, E, C in relation to the risk of bladder cancer in the ATBC cohort study. Br J Cancer 2002; 87: 960–965. 12434284 10.1038/sj.bjc.6600604 PMC2364321

[7] Aggarwal BB, Sundaram C, Prasad S, Kannappan R. Tocotrienols, the vitamin E of the 21st century: Its potential against cancer and other chronic diseases. Biochem Pharmacol. 2010; 80: 1613–1631. 10.1016/j.bcp.2010.07.043 20696139 PMC2956867

[8] Sen CK, Khanna S, Rink C, Roy S. Tocotrienols: The Emerging Face of Natural Vitamin E. Vitam Horm. 2007; 76: 203–261. 17628176 10.1016/S0083-6729(07)76008-9 PMC3681510

[9] Kannappan R, Gupta S, Kim J, Aggarwal BB. Tocotrienols fight cancer by targeting multiple cell signaling pathways. Genes Nutr. 2012; 7: 43–52. 10.1007/s12263-011-0220-3 21484157 PMC3250528

[10] Lee IM, Cook NR, Gaziano JM, Gordon D, Ridker PM, Manson JE, et al Vitamin E in the primary prevention of cardiovascular disease and cancer: the Women's Health Study: a randomized controlled trial. JAMA 2005; 294: 56–65. 15998891 10.1001/jama.294.1.56

[11] Lippman SM, Klein EA, Goodman PJ, Lucia MS, Thompson IM, Ford LG, et al Effect of selenium and vitamin E on risk of prostate cancer and other cancers: the Selenium and Vitamin E Cancer Prevention Trial (SELECT). JAMA 2009; 301: 39–51. 10.1001/jama.2008.864 19066370 PMC3682779

[12] Miyazawa T, Shibata A, Sookwong P, Kawakami Y, Eitsuka T, Asai A, et al Antiangiogenic and anticancer potential of unsaturated vitamin E (tocotrienol). J Nutr Biochem. 2009; 20: 79–86. 10.1016/j.jnutbio.2008.09.003 19071006

[13] Shin-Kang S, Ramsauer VP, Lightner J, Chakraborty K, Stone W, Campbell S, et al Tocotrienols inhibit AKT and ERK activation and suppress pancreatic cancer cell proliferation by suppressing the ErbB2 pathway. Free Radic Biol Med. 2011; 51: 1164–1174. 10.1016/j.freeradbiomed.2011.06.008 21723941

[14] Yap WN, Zaiden N, Tan YL, Ngoh CP, Zhang XW, Wong YC, et al Id1, inhibitor of differentiation, is a key protein mediating anti-tumor responses of gamma-tocotrienol in breast cancer cells. Cancer Lett. 2010; 291: 187–199. 10.1016/j.canlet.2009.10.012 19926394

[15] Husain K, Francois RA, Yamauchi T, Perez M, Sebti SM, Malafa MP. Vitamin E δ-Tocotrienol Augments the Antitumor Activity of Gemcitabine and Suppresses Constitutive NF-κB Activation in Pancreatic Cancer. Mol Cancer Ther. 2011; 10: 2363–2372. 10.1158/1535-7163.MCT-11-0424 21971120 PMC3237822

[16] Zhang JS, Li DM, He N, Liu YH, Wang CH, Jiang SQ, et al A paraptosis-like cell death induced by δ-tocotrienol in human colon carcinoma SW620 cells is associated with the suppression of the Wnt signaling pathway. Toxicology 2011; 285: 8–17. 10.1016/j.tox.2011.03.011 21453743

[17] Pierpaoli E, Viola V, Pilolli F, Piroddi M, Galli F, Provinciali M. Gamma- and delta-tocotrienols exert a more potent anticancer effect than alpha-tocopheryl succinate on breast cancer cell lines irrespective of HER-2/neu expression. Life Sci. 2010; 86: 668–675. 10.1016/j.lfs.2010.02.018 20188744

[18] Yu H, Pardoll D, Jove R. STATs in cancer inflammation and immunity: a leading role for STAT3. Nat Rev Cancer 2009; 9:798–809. 10.1038/nrc2734 19851315 PMC4856025

[19] Xiong A, Yang Z, Shen Y, Zhou J, Shen Q. Transcription Factor STAT3 as a Novel Molecular Target for Cancer Prevention. Cancers 2014; 6:926–957. 10.3390/cancers6020926 24743778 PMC4074810

[20] Pathania AS, Kumar S, Guru SK, Bhushan S, Sharma PR, Aithagani SK, et al The synthetic tryptanthrin analogue suppresses STAT3 signaling and induces Caspase dependent apoptosis via ERK up regulation in human leukemia HL-60 cell. PLoS One 2014; 9:e110411 10.1371/journal.pone.0110411 25383546 PMC4226462

[21] Siveen KS, Sikka S, Surana R, Dai X, Zhang J, Kumar AP, et al Targeting the STAT3 signaling pathway in cancer: role of synthetic and natural inhibitors. Biochim Biophys Acta 2014; 1845:136–154. 10.1016/j.bbcan.2013.12.005 24388873

[22] Ho PL, Lay EJ, Jian W, Parra D, Chan KS. Stat3 activation in urothelial stem cells leads to direct progression to invasive bladder cancer. Cancer Res. 2012; 72: 3135–3142. 10.1158/0008-5472.CAN-11-3195 22532166 PMC3418917

[23] Dong Y, Lu B, Zhang X, Zhang J, Lai L, Li D, et al Cucurbitacin E, a tetracyclic triterpenes compound from Chinese medicine, inhibits tumor angiogenesis through VEGFR2-mediated Jak2–STAT3 signaling pathway. Carcinogenesis 2010; 31: 2097–2104. 10.1093/carcin/bgq167 20732905

[24] Guo S, Sun F, Guo Z, Li W, Alfano A, Chen H, et al Tyrosine Kinase ETK/BMX Is Up-Regulated in Bladder Cancer and Predicts Poor Prognosis in Patients with Cystectomy. PLoS One 2011; 6: e17778 10.1371/journal.pone.0017778 21408190 PMC3049795

[25] Neri B, Doni L, Fulignati C, Gemelli MT, Turrini M, Di Cello V, et al Gemcitabine plus Epi-doxorubicin as the first-line chemotherapy for bladder cancer in advanced or metastatic stage: a phase II. Anticancer Res. 2002; 22: 2981–2984. 12530029

[26] Yafi FA, North S, Kassouf W. First- and second-line therapy for metastatic urothelial carcinoma of the bladder. Curr Oncol. 2011; 18: e25–34. 21331269 10.3747/co.v18i1.695 PMC3031362

[27] Ji X, Wang Z, Sarkar FH, Gupta SV. Inhibition of cell growth and induction of apoptosis in non-small cell lung cancer cells by delta-tocotrienol is associated with notch-1 down-regulation. J Cell Biochem. 2011; 112: 2773–2783. 10.1002/jcb.23184 21598300

[28] Chen CL, Cen L, Kohout J, Hutzen B, Chan C, Hsieh FC, et al Signal transducer and activator of transcription 3 activation is associated with bladder cancer cell growth and survival. Mol Cancer 2008; 7: 78 10.1186/1476-4598-7-78 18939995 PMC2577686

[29] Chan KS, Espinosa I, Chao M, Wong D, Ailles L, Diehn M, et al Identification, molecular characterization, clinical prognosis, and therapeutic targeting of human bladder tumor-initiating cells. Proc Natl Acad Sci USA. 2009; 106: 14016–14021. 10.1073/pnas.0906549106 19666525 PMC2720852

[30] Itoh M, Murata T, Suzuki T, Shindoh M, Nakajima K, Imai K, et al Requirement of STAT3 activation for maximal collagenase-1 (MMP-1) induction by epidermal growth factor and malignant characteristics in T24 bladder cancer cells. Oncogene 2006; 25: 1195–1204. 16205632 10.1038/sj.onc.1209149

[31] Holopainen T, López-Alpuche V, Zheng W, Heljasvaara R, Jones D, He Y, et al Deletion of the endothelial Bmx tyrosine kinase decreases tumor angiogenesis and growth. Cancer Res. 2012; 72:3512–3521. 10.1158/0008-5472.CAN-11-1070 22593188

[32] Jiang X, Borgesi RA, McKnight NC, Kaur R, Carpenter CL, Balk SP. Activation of nonreceptor tyrosine kinase Bmx/Etk mediated by phosphoinositide 3-kinase, epidermal growth factor receptor, and ErbB3 in prostate cancer cells. J Biol Chem. 2007; 282:32689–32698. 10.1074/jbc.M703412200 17823122

[33] Tsai YT, Su YH, Fang SS, Huang TN, Qiu Y, Jou YS, et al Etk, a Btk Family Tyrosine Kinase, Mediates Cellular Transformation by Linking Src to STAT3 Activation. Mol Cell Biol. 2000; 20: 2043–2054. 10688651 10.1128/mcb.20.6.2043-2054.2000 PMC110821

[34] Guryanova OA, Wu Q, Cheng L, Lathia JD, Huang Z, Yang J, et al Nonreceptor tyrosine kinase BMX maintains self-renewal and tumorigenic potential of glioblastoma stem cells by activating STAT3. Cancer Cell 2011; 19: 498–511. 10.1016/j.ccr.2011.03.004 21481791 PMC3076106

[35] Oka T, Ouchida M, Koyama M, Ogama Y, Takada S, Nakatani Y, et al Gene silencing of the tyrosine phosphatase SHP1 gene by aberrant methylation in leukemias/lymphomas. Cancer Res. 2002; 62: 6390–6394. 12438221

[36] Kannappan R1, Yadav VR, Aggarwal BB. γ-Tocotrienol but not γ-tocopherol blocks STAT3 cell signaling pathway through induction of protein-tyrosine phosphatase SHP-1 and sensitizes tumor cells to chemotherapeutic agents. J Biol Chem. 2010; 285:33520–33528. 10.1074/jbc.M110.158378 20720018 PMC2963373

[37] Rajendran P, Li F, Manu KA, Shanmugam MK, Loo SY, Kumar AP, et al γ-Tocotrienol is a novel inhibitor of constitutive and inducible STAT3 signalling pathway in human hepatocellular carcinoma: potential role as an antiproliferative, pro-apoptotic and chemosensitizing agent. Br J Pharmacol. 2011; 163:283–298. 10.1111/j.1476-5381.2010.01187.x 21198544 PMC3087132

[38] Kanai K, Kikuchi E, Mikami S, Suzuki E, Uchida Y, Kodaira K, et al Vitamin E succinate induced apoptosis and enhanced chemosensitivity to paclitaxel in human bladder cancer cells in vitro and in vivo. Cancer Sci. 2010; 101: 216–223. 10.1111/j.1349-7006.2009.01362.x 19824995 PMC11158940

