# Supplementary figures and images for: δ-Tocotrienol Induces Human Bladder Cancer Cell Growth Arrest, Apoptosis and Chemosensitization through Inhibition of STAT3 Pathway
Source: PLoS One. 2015 Apr 7;10(4):e0122712. doi: 10.1371/journal.pone.0122712 (PMC4388509; doi:10.1371/journal.pone.0122712)

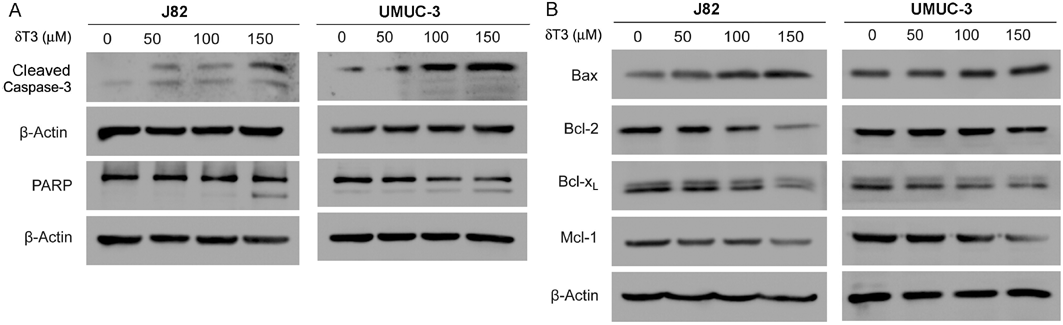

Supplement: S1 Fig — The cleavage of Caspase-3 and PARP (A), as well as the induction of pro-apoptotic Bax protein level and reduction of anti-apoptotic Bcl-2, BclxL and Mcl-1 protein levels were detected in J82 and UMUC-3 cells, upon the δ-T3 treatment for 24 h. β-Actin was used as the loading control. (TIF) [file pone.0122712.s001.tif]

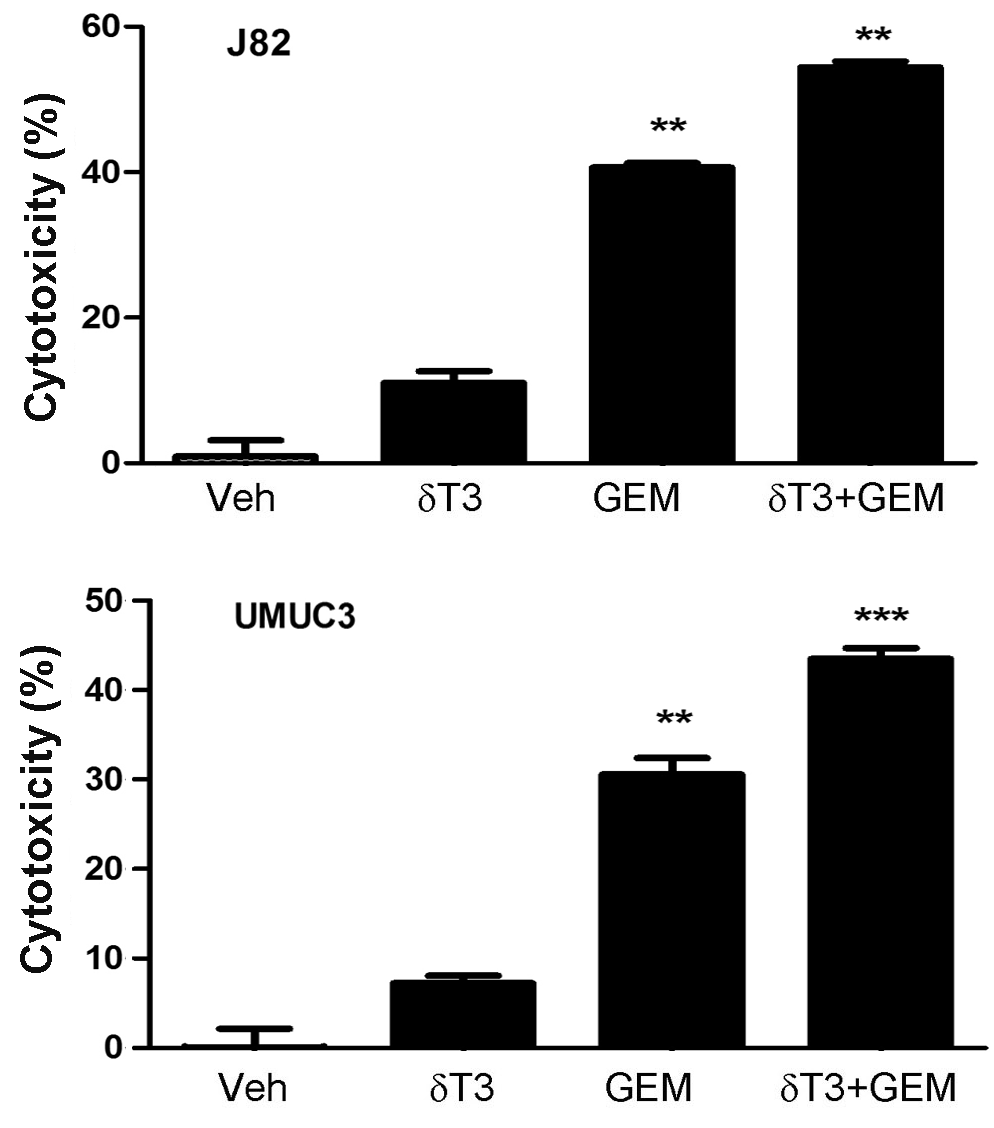

Supplement: S2 Fig — J82 and UMUC-3 cells were incubated for 48 h in the presence of 25 μM δ-T3 and/or 0.08 μM GEM. Then, the percentage of cell viability was determined by MTT assay. **, P < 0.01; ***, P < 0.001. (TIF) [file pone.0122712.s002.tif]
